# Impacts of farm to school on children's perception of food

**DOI:** 10.1007/s44403-025-00020-5

**Published:** 2025-03-27

**Authors:** Lesley Leake, Maria McClam, Amanda Howell, Katie Schreiber

**Affiliations:** 1 Arnold School of Public Health, University of South Carolina, 220 Stoneridge Dr. Suite 103, Columbia, SC 29210 USA; 2 College of Charleston, Charleston, SC USA

**Keywords:** Farm to school, Childhood nutrition, Rich pictures, Qualitative methods

## Abstract

**Objective:**

To evaluate the impact of a farm to school program on urban, elementary-aged children.

**Methods:**

A classroom of students receiving a farm to school intervention was compared to a control classroom at the same school. Pre-and post-surveys measured knowledge, attitudes, and consumption of fruits and vegetables in both groups. Dietary recalls measured consumption of fruits and vegetables post-intervention. Rich pictures captured children’s mental model of food post intervention.

**Results:**

The rich pictures of farm to school students were more likely to include fruits and vegetables (OR = 7.2; 95% CI = 1.66,31.26) and imagery of the natural environment (insignificant) compared to the control group. Their overall mental models of food were significantly healthier (*p* < .01). No students in either group (*n* = 41) ate the USDA recommended servings of fruits and vegetables.

**Conclusions and implications:**

Farm to school programs influence children’s mental model of food and may give students a more robust understanding of where food comes from.

## Introduction

Consumption of minimally processed fruits and vegetables (F&V) protects against cancer [1–4], cardiovascular disease [4], obesity [3], type II diabetes, low bone density [5], and depression [6], by supplying our bodies with vitamins, minerals, dietary fiber, and phytochemicals [7]. Yet in the United States (US), 60% of children do not meet the daily recommended fruit intake, and 93% of children do not eat enough vegetables [8].

This chronic under-consumption of F&V contributes to an alarming childhood obesity rate in the US [9, 10]; a staggering 1 in 5 children are obese [11]. Childhood obesity is a predictor of obesity lasting into adulthood [12], an outcome costing the US millions of lives [13] and billions of dollars in healthcare costs each year [14]. Interventions that increase F&V consumption can support children’s health [15]; one of these potential interventions is Farm to School (F2S).

F2S is a community-based intervention that uses partnerships between schools, community-based organizations, and local farms to connect school children with fresh and locally grown produce [16]. The F2S movement began in the 1990s as parents, farmers, and schools across the country tested ways to provide school children with more fresh and local food options in school [16]. For example, a parent in a California school tested the idea of a “Farmer’s Market Salad Bar” made up of produce from local farms to see if students would eat more fresh produce—and they did. Around the same time, a farm cooperative in northern Florida began selling produce to the nearby school district [16]. Demand for the produce grew over time and after ten years the farm cooperative was selling produce to seventy-two school districts in the region and feeding one million children with their produce [16]. As the F2S movement grew, efforts became more coordinated and a nation-wide Farm to School Network took shape [16]. The Farm to School Network is a hub for communities working to bring local food sourcing and agriculture education into school systems by providing information, advocacy, and networking [17]. By 2010, the Farm to School Network gained national attention and, under the Obama administration, the US Congress passed the Healthy, Hunger-Free Kids Act which included $40 million to support F2S efforts [16].

According to the Farm to School Network, F2S programs include three components: school gardens, education, and procurement of local foods [17], and can include any combination of other strategies including composting and recycling initiatives, farm tours, taste testing, cooking classes, and changes to the school cafeteria [16]. Because those schools using a F2S approach are free to include whatever strategies best meet their needs and capacity, F2S program evaluation outcomes vary, and there is the need for continual evaluation. However, evaluation findings generally indicate that F2S programs can increase school meal participation, nutritious food selection, knowledge of nutrition and agriculture, willingness to try new foods, and, in some cases, F&V consumption [18, 19].

The extent to which F2S programs increase actual F&V consumption is inconsistent; several systematic literature reviews of F2S evaluations indicate a range of effectiveness of F2S program evaluations in promoting increased F&V consumption [18–20]. A 2020 systematic literature review conducted by Prescott et al. of twenty-one F2S program evaluations—some explicitly called F2S and others consisting of F2S components but without the explicit F2S name—found that programs consistently showed positive impacts on nutrition-related knowledge but impacts on F&V consumption were inconsistent [19]. While the ultimate goal of most F2S programs is to improve children’s dietary behavior, intrapersonal characteristics like knowledge, attitudes, and intentions are a predictor of behavior according to the theory of planned behavior [21], health belief model [22], and social cognitive theory [23].

The Green Heart Project (GHP), located in Charleston, South Carolina implements a robust F2S program for elementary and middle school aged students which utilizes experiential learning techniques to enhance youths’ understanding of healthy eating and environmental sustainability. GHP’s *Classic Curriculum* is provided to elementary-school-aged (typically ages 6–11 years old) students over the course of either a trimester (about 9 sessions), a semester (about 15 sessions), or a school year (about 30 sessions) and includes hands on gardening, produce taste-tests, and nutrition, science, and environmental stewardship education. For example, GHP’s curriculum includes a “Strawberry Planting” lesson which teaches students how strawberries grow from a seed into a fruit plant. They learn the basic anatomy of a strawberry plant and the nutrients necessary for the plan to thrive, and they receive hands-on experience in the garden planting strawberry starts. The overarching goals of the lesson are for students to understand the lifecycle of F&V from a seed to the version we see on our plates, for the students to have a role in growing and preparing this food, and for students to understand the health benefits of F&V.

The purpose of this study was to evaluate the impact that GHP’s yearlong F2S program had on children’s knowledge of, attitudes towards, and consumption of F&V.

## Methods

### Study design

To evaluate the impact that GHP’s yearlong F2S program had on children’s knowledge of, attitudes towards, and consumption of F&V, researchers implemented a pre-post study design with a control group among elementary school students at an independent school in the 2022–2023 school year. Mixed method data collection included a parent survey to assess student demographics, a pre-intervention survey assessing dietary behavior and attitudes, a post-intervention survey assessing dietary behavior and attitudes, post-intervention 24-h dietary recall interviews, and a post-intervention rich picture drawing activity. The school’s entire sixth grade class (approximately ages 11–12 years old) served as the intervention group (*n* = 25) in the 2022–2023 school year; the entire fifth grade class (approximately ages 10–11 years old) was selected as a naturalistic control (*n* = 16) group. The control class had a delayed intervention and received GHP’s F2S program in the 2023–2024 school year.

### Participants and recruitment

Fifth and 6th grade students and their families from the independent school were invited to participate in our study. All students and families were given the opportunity to opt out of data collection activities and youth assent was obtained before data collection. The data collection activities used in this F2S evaluation were approved under exempt status by the Institutional Review Board at the University of South Carolina.

### Instruments, measures, and procedures

In the fall of 2022, 5th and 6th grade classroom teachers at the independent school invited parents of students to participate in an online demographic survey. This parent survey captured the child’s grade, race, zip code, and annual household income and was used to assess any demographic differences between the intervention and control groups (Table 2).

Additionally, pre-intervention surveys (*n* = 40) were completed with the intervention (6th grade, *n* = 24) and control (5th grade, *n* = 16) classrooms during the fall of 2022; post-intervention surveys (*n* = 41; 6th grade/intervention = 25, 5th grade/control = 16,) were completed with both groups at the end of the spring 2023 semester. Pre- and post-surveys assessed a) attitudes towards F&V, b) F&V consumption, and c) attitudes towards environmental stewardship, as well as several program evaluation questions. Example survey questions included: “*Rate how you feel about eating fruits and veggies*.”, “*How often do you eat fruits and veggies?*”, and*”Did you share anything you learned during* GHP *lessons with your family*?”. Most but not all constructs on the surveys were assessed using a 5-point Likert scale.

At the end of the 2022–2023 school year, in May 2023, the intervention and control classrooms participated in two data collection activities while at school during their respective science classes. The research team conducted a rich picture drawing activity (*n* = 39)^1^ and individual 24-h dietary recall interviews (*n* = 41) with all students in the intervention and control classrooms, respectively. Rich pictures are drawings that depict a “map” of a system or environment. In addition to representing an external reality, rich pictures are a tool to generate reflection and conversation that can reveal new insights; they have been used by researchers to understand children’s mental model of mental phenomenon [24]. During the rich picture activity, researchers asked students to draw their responses to a series of appreciative inquiry questions about food (e.g., *draw the first thing that comes to mind when you think of food, what foods do you eat*). After all drawings were finished, students were invited to explain their answers to the group and discuss similarities and differences between their perspectives.

Following the rich picture activity, students participated in individual 24-h dietary recall interviews. While there are multiple methods for assessing dietary behavior over a period of time, the 24-h recall method is recommended by the NIH for examining total diet over a short period of time as was done in this study [25]. The 24-h dietary recall interview protocol was adapted from Baxter et al. [26], who validated the protocol for use with students as young as fourth grade [26]. Students also completed a written food diary for the 24-h prior to the interview to enhance their recall during the interview and reduce recall bias. This food diary was provided to students by their teacher the day before the interviews.

Per the Baxter et al. protocol [26] the interview first asked students to remember food consumed that day in chronological order (e.g., the current day’s breakfast, snack, lunch) then the day before in chronological order to complete the 24-h cycle (e.g., yesterday’s lunch, after-lunch snack, dinner, etc.). Interviews captured mealtime, food item, quantity, and brand of food item (when appropriate). Interviewers used students’ food diaries to corroborate recall and to probe further when there were discrepancies or clarity was needed. In addition to food recall questions, interviews also included questions like “*Is this similar to what you would normally eat?*” and “*In general, do you eat at least 2 fruits a day?*”.

### Data analysis

Parent surveys were analyzed using *Microsoft Excel for Mac* Version 16.86 (Microsoft Corporation) for descriptive statistics. All pre- and post-survey data were analyzed using *SAS* version 9.4 (SAS Institute Inc). Basic descriptive and inferential statistics were calculated for survey responses by group (intervention vs control). Independent sample t-tests compared the intervention and control groups’ responses to each question on the post-intervention surveys. Researchers also used independent sample t-tests to compare pre-intervention survey responses to post-intervention survey responses for both groups. Given that the data was positively skewed and not all assumptions of normality were met, researchers also calculated differences using a Wilcoxon Mann Whitney test but found no difference in results.

In addition to detailed note taking during data collection, rich picture activities and all interviews were audio recorded and transcribed. Rich pictures were analyzed both deductively and inductively. For the deductive analysis, five reviewers independently scored each rich picture on three dimensions (healthy food, environment, and impacts on the physical body) using a 5-point scale (Table 1) adopting methods used by Brauner et al. [24]. The five reviewers’ ratings of each dimension were averaged, and the average scores were compared between intervention and control groups using a two-sample t-test in *Microsoft Excel for Mac* Version 16.86 (Microsoft Corporation). An Intraclass Correlation Coefficient was calculated in *SPSS Statistics* version 29 (IBM Corporation) to evaluate how consistent the reviewers’ ratings were to each other, or the inter-rater reliability. Additionally, two researchers independently conducted a conventional content analysis to derive themes from the drawings [27] and to count the number of instances each derived theme was present [27]. Finally, based on a grounded theory framework, the researchers compared their conventional and summative content analysis prior to the final advanced coding where all analyses were synthesized [28]. This inductive coding technique was used to elucidate any other themes in the rich pictures beyond the original three dimensions of healthy food, environment, and impacts on the physical body [28].

**Table 1:** Dimensions and scale of rich picture deductive analysis

Dimensions	Scale
1. The drawings in these boxes display healthy food (e.g. whole, minimally processed foods or a nutritious meal) 2. The drawings in this box portray images of the environment (e.g. gardening, farming, and/or the natural environment) 3. The drawing in this box shows an understanding of how food is linked to health and impacts the physical body	1-Strongly Disagree 2-Disagree 3-Neutral 4-Agree 5-Strongly Agree

Two researchers independently coded the 24-h dietary recall portion of all interviews using a summative approach [27] to count the servings of F&V consumed by each participant. The US Department of Agriculture (USDA) MyPlate guidelines (www.MyPlate.gov) were used to identify F&V and to calculate the serving size of a fruit or vegetable (e.g., avocado is considered a vegetable on MyPlate and the serving size is 1 whole avocado). If a food item quantity was reported as a range (e.g., 2–3 strawberries), the average was used (e.g. 2.5 strawberries). If an identifiable brand product was listed (e.g., 25 Goldfish crackers) the actual serving size was taken from the online food label (e.g., Goldfish label says serving size is 55 crackers). Researchers 1 and 2 convened with a third researcher to develop consensus on servings of F&V for each child; the third researcher was consulted to resolve any discrepancies between serving sizes assigned by Researchers 1 and 2. In addition to calculating servings of F&V consumed by each participant, researchers also compared the number of students who met the USDA recommendations for eating two fruits and three vegetables a day using an odds ratio and 95% confidence interval. Finally, all open-ended interview questions were analyzed inductively using qualitative analysis software, Dedoose.

During the preparation of this manuscript the authors used ChatGPT to improve readability. After using this tool, the authors reviewed and edited the content as needed and take full responsibility for the content of the publication.

## Results

### Study participants

Intervention and control classrooms were demographically similar. In both groups, most respondents were White (75%), had an annual household income of $100,000 to $200,000 (45%), and lived in a variety of zip codes in the Charleston area. In addition, all parents (100%) reported they loved eating F&V (rated 5 on a 5-point Likert scale). There were no statistically significant differences in race/ethnicity, household income, or zip code between groups (Table 2).

**Table 2:** Parent demographic survey

	Total (*n* = 20)	Intervention (*n* = 11)	Control (*n* = 9)	*p*-value^2^
	n (%)^a^	n (%)^a^	n (%)^a^	
Race/ethnicity				0.85
White	15 (75)	9 (81)	6 (67)	
Black or African American	2 (10)	1 (9)	1 (11)	
Black, Hispanic/Latino	2 (10)	1 (9)	1 (11)	
Prefer not to say	1 (5)	0 (0)	1 (11)	
Annual household income				0.91
More than $200,000	6 (30)	4 (36)	2 (22)	
$100,000—$200,000	9 (45)	5 (45)	4 (44)	
$50,000—$100,000	3 (15)	1 (9)	2 (22)	
Prefer not to say	2 (10)	1 (9)	1 (11)	
Zip Code				0.55
Other	7 (35)	4 (36)	3 (33)	
*redacted*	4 (20)	3 (27)	1 (11)	
*redacted*	3 (15)	1 (9)	2 (22)	
*redacted*	2 (10)	0 (0)	2 (22)	
*redacted*	2 (10)	2 (18)	0 (0)	
*redacted*	2 (10)	1 (9)	1 (11)	

^a^Percents that do not equal 100 are because of rounding

^2^*P*-values represented are from chai squared tests

### Rich pictures

**Fig. 1:**
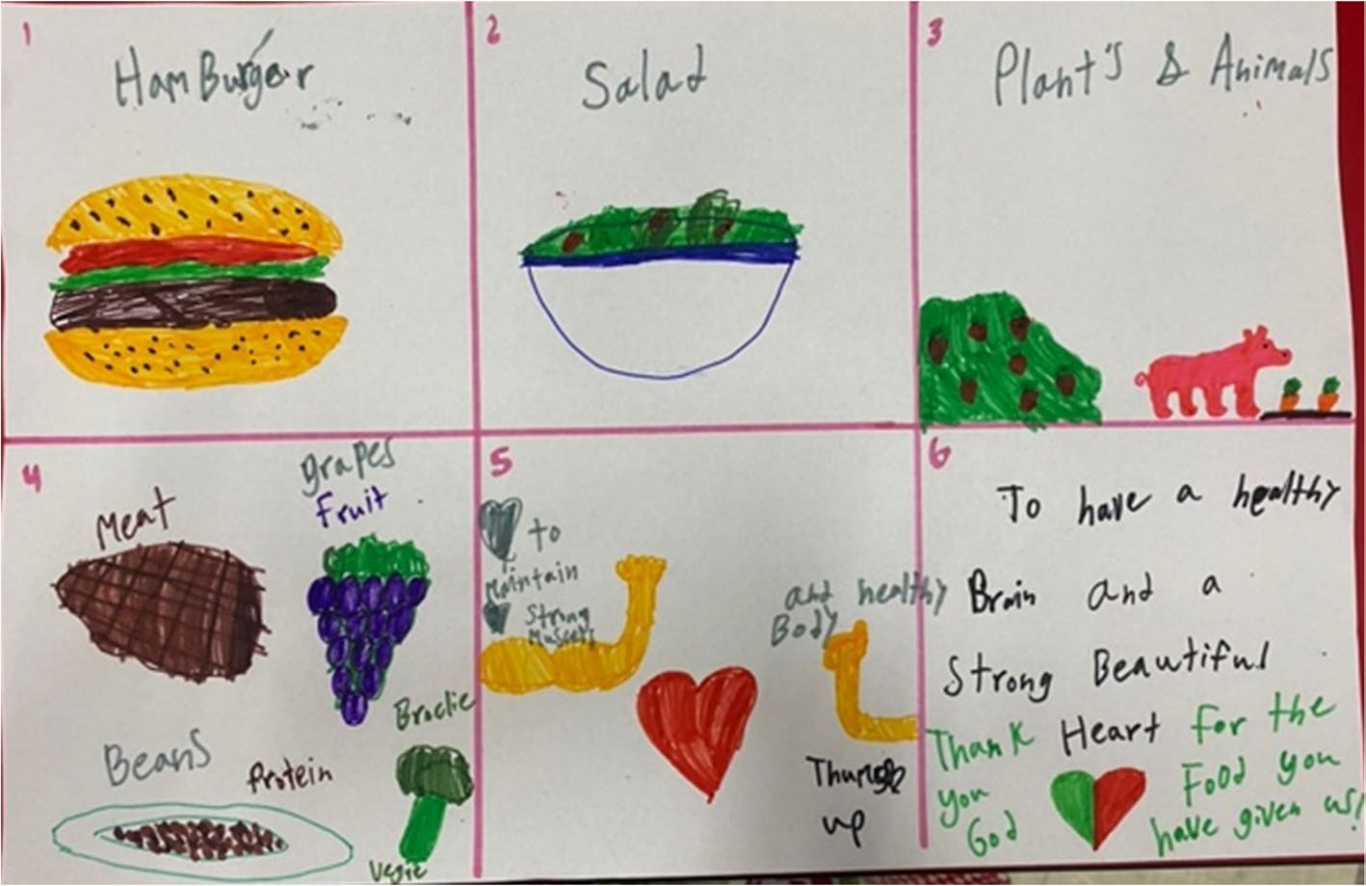
Example of a student’s rich picture drawing

In total, thirty-nine rich pictures from fourteen 5th graders (control group) and twenty-five 6th graders (intervention group) were completed (Fig. 1). Common foods that appeared in most drawings across both groups included carrots, apples, strawberries, chicken, fish, burgers, pizza, and pasta. Both groups appeared to understand healthy food; when asked to draw *What do you think of when you think of healthy food?* most drew or described nutrient rich foods including “*protein*”, “*grains*”, “*fruits*”, “*vegetables*”, carrots, and apples. When asked to draw why they eat food, most participants across both groups drew some characteristics of physical health like strong muscles, organs, nutrients, heart health, or brain health. Finally, both groups’ rich pictures depicted positive attitudes towards food like smiley faces, hearts, or thumbs up.

Rich pictures indicated two key differences between the intervention and control groups. First, when asked “*what foods do you eat?*”, the intervention group was more likely to draw F&V; approximately 80% of intervention participants drew predominately F&V compared to 36% of control participants. The odds of drawing F&V were significantly higher in the intervention group compared to those in the control (OR = 7.2; 95% CI = 1.66, 31.26). Second, when asked “*where does your food come from?*”, the intervention group was slightly more likely to draw images of nature (e.g., trees, farms, cows, earth, gardens); 88% of intervention participants illustrated natural elements compared to 71% of control participants. The odds of drawing nature were higher in the intervention group compared to those in the control (OR = 2.93; 95% CI = 0.55, 15.63) but not significantly higher.

Results from the deductive Rich Picture analysis, in which five reviewers rated each Rich Picture on 3 dimensions (healthy food, environment, and impacts on the physical body), revealed the intervention group had a higher average score on all three dimensions compared to the control group. However, only Dimension 1 (Healthy Food, defined as “whole, minimally processed foods or a nutritious meal*”*) was statistically significantly different (two sample t-test; *p* < 0.01); students who received the GHP F2S program were significantly more likely to draw images of healthy food in their rich pictures (Table 3). To assess inter-rater reliability between the 5 reviewers, researchers calculated an Intraclass Correlation Coefficient (two-way fixed model, absolute agreement type) for average measures (ICC(1,k) = 0.890) which was statistically significant (*p* < 0.001) and indicates good inter-rater reliability between the five reviewers.

**Table 3:** Average rich picture score by group for 3 key dimensions

	Intervention	Control	*p*-value^1^
Dimension 1: Healthy Food	4.04	3.43	> 0.01*
Dimension 2: Environment	3.7	3.53	0.73
Dimension 3: Impacts on Physical Body	3.81	3.55	0.33

^1^*P*-values represented are from chai squared tests

^*^Statistically significant and corresponding *p*-value < 0.05

### 24-h Dietary recall interviews

A total of 41 students from intervention (*n* = 25) and control (*n* = 16) classrooms participated in 24-h dietary recall interviews. Students in the intervention group consumed more F&V on average, but the difference was insignificant. Intervention students reported consuming an average of 1.5 servings of fruit and 0.9 servings of vegetables during the 24-h recall period. Respectively, students in the control group reported consuming an average of 1.1 servings of fruit and 0.8 servings of vegetables. Forty percent (40%) of students in the intervention group ate at least 2 servings of fruit, compared to 19% of students in the control group; twelve percent (12%) of students in the intervention group ate at least 2.5 servings of vegetables compared to 0% of the control group (Table 4). The odds of eating recommended servings of both F&V was higher in the intervention group compared to those in the control (OR for fruit = 2.89; 95% CI = 0.65,12.80; OR for vegetables = 5.13; 95% CI = 0.25, 106.30) but not significantly higher.

**Table 4:** Number of students who reported consuming the daily recommended amounts of fruits and vegetables based on US dietary guidelines

	Total (*n* = 41)	Intervention (*n* = 25)	Control (*n* = 16)
	n (%)	n (%)	n (%)
Met the US dietary guidelines for fruit (2 servings)	13 (32)	10 (40)	3 (19)
Met the US dietary guidelines for vegetables (2.5 servings)	3 (7)	3 (12)	0 (0)

No student in either group met the daily recommended amounts of fruits *and* vegetables, which is two servings of fruit and 2.5 servings of vegetables for children 9–13 years old [29]. Looking across both groups, students ate more fruit (mean = 1.4 servings) compared to vegetables (mean = 0.9 servings) and the odds of meeting the USDA recommendation for servings of fruit was higher than that of vegetables (OR = 5.88; 95% CI = 1.53, 22.62). While no students met the recommendation for fruit *and* vegetable consumption, some participants did consume the recommend two servings of fruits (*n* = 13) *or* 2.5 servings of vegetables (*n* = 3) respectively.

Most students across both the control (88%) and intervention groups (96%) said the food they reported on their 24-h dietary recall was similar to what they normally eat. The few (*n* = 3) students who indicated their 24-h recalls were not reflective of their “normal” diet said this was because they were sick (*n* = 2) or had gotten braces put on (*n* = 1). Other students noted particular items that may not be included in their everyday diet: *“I only have donuts for breakfast on a special occasion”, “I would usually eat an apple instead of two bags of chips”, “I don’t always eat Lunchables.”*

At the end of the interviews, participants were asked to think about whether they generally consume two servings of fruit and three servings of vegetables a day. Contradictory to students’ dietary recalls, students in both groups perceived they generally eat at least two fruits a day (control: *n* = 12, 75%; intervention: *n* = 16, 70%) or three vegetables a day (control: *n* = 9, 56%; intervention: *n* = 11, 48%); this indicates students think they eat more F&V a day than their recalls show.

### Pre- and post-intervention surveys

A total of 40 pre-intervention surveys (control *n* = 16, intervention *n* = 24) and 41 post-intervention surveys (control *n* = 16, intervention *n* = 25) were completed. Pre-surveys indicate the two groups (intervention and control groups) were comparable at baseline. There were no significant differences between the two groups at baseline except in one category; the intervention group had a statistically higher mean score for *confidence towards cooking* at baseline compared to the control group. Among the intervention group, or students who received GHP’s curriculum, attitudes towards F&V, gardening, and the environment as well as their dietary behavior improved from pre- to post-surveys (Table 5). Post-surveys had more favorable responses among the intervention group compared to the control group for questions “*If you grew fruit and vegetables, would you be more likely to eat them?”,* “*I feel comfortable working in a garden.”, and “How often do you eat fruits and veggies?*” (Table 6). These results were insignificant, likely due to our small sample size.

**Table 5:** Mean survey scores for intervention group comparing pre to post

	Pre Survey (*n* = 24) Mean	Post Survey (*n* = 25) Mean	*p*-value^1^
Rate how you feel about eating fruits and veggies	4.00	4.00	1
Rate how you feel about trying new foods	3.75	3.76	0.97
If you grew fruit and vegetables, would you be more likely to eat them?	3.96	4.24	0.33
I feel comfortable working in a garden	4.42	4.56	0.45
I feel confident taking care of the environment	4.38	4.56	0.37
I feel confident cooking a meal	3.75	4.04	0.42
How often do you eat fruits and veggies? (Number per day)	3.51	2.72	0.10

^1^*p*-value is from a two independent samples *t*-test

**Table 6:** Mean survey scores for post survey data comparing groups

	Intervention (*n* = 25) Mean	Control (*n* = 16) Mean	*p*-value^1^
Rate how you feel about eating fruits and veggies	4	4.41	0.07
Rate how you feel about trying new foods	3.76	3.97	0.12
If you grew fruit and vegetables, would you be more likely to eat them?	4.24	4.13	0.67
I feel comfortable working in a garden	4.56	4.38	0.40
I feel confident taking care of the environment	4.56	4.63	0.77
I feel confident cooking a meal	4.04	4.19	0.64
How often do you eat fruits and veggies? (Number per day)	2.72	2.50	0.70

^1^*p*-value is from a two independent samples t-test

## Discussion

F2S programs connect school children with fresh and locally grown produce, impacting their nutrition related knowledge and behavior. Overall, our findings indicated that the F2S program implemented by GHP had a positive impact on students, although only some findings were significant. The most significant impact seems to be on students’ mental model of food, which is consistent with prior research findings [30]. When asked to contemplate various questions related to food (e.g., what is healthy food, favorite foods, why we eat, importance of food) students receiving the F2S intervention were significantly more likely to draw fruits, vegetables, and “healthy food” than the control group. This indicates students who participate in F2S may have a healthier mental model of food; when they think of food, they are more likely to think of F&V.

F2S programs may also give students a more robust understanding of where food comes from. Fundamental components of F2S programming include hands on gardening, plant science education, and procurement of local farm-sourced produce [17], which can help children understand where their food comes from. Our study found that F2S students’ mental model of food included more imagery of the natural environment (e.g., farms, fields, trees, gardens) compared to the control group who drew images of groceries stores, convenience stores, and big box stores.

Dietary recall data did not reveal statistically significant differences in F&V consumption between groups. This is consistent with a systematic literature review of twenty-one F2S style programs, which found that F2S programs consistently show positive impacts on nutrition-related knowledge but changes in actual F&V consumption varies [19]. While the ultimate goal of many F2S programs is to increase F&V consumption, behavioral theories and prior research indicate that changing knowledge, attitudes, and perceptions can lead to behavior change [21–23].

A secondary finding in this study is that no students reported eating the recommended daily servings of F&V. This is also consistent with prior research. The World Health Organization recommends children and adults consume 400 g or five total servings of F&V each day [31]. Many children across the globe are not consuming the recommended quantity of F&V. An analysis of global school-based survey data found that on average 12- to 17-year-olds consume 1.43 servings of fruit a day and 1.75 servings of vegetables a day—almost 2 servings short of the World Health Organization recommendation [32]. In the United States, it is reported that 60% of children do not meet the daily recommended fruit intake, and 93% of children do not eat enough vegetables [8]. Older children appear to be the least likely to meet the recommended intake of F&V. On average children 14- to 18-years-old eat one serving of fruit and one serving of vegetables per day [33]. Of particular interest to this study is the 9–13 age group. Within this age group the guidelines vary greatly based on sex and physical activity level, but generally speaking, children in this age range need 2 servings of fruit and 2.5 servings of vegetables daily. The average F&V consumption for 9- to 13-year-olds of both sexes falls below recommended levels; children in this age range are consuming approximately one serving of fruit and one serving of vegetables each day [29].

One explanation for this lack of F&V consumption in the US is that students believe they are eating more than they do. When asked *In general, do you eat at least 2 fruits/3 servings of vegetables a day?* (the general non-age-specific recommendation) most study participants said “yes”; however, no participants actually consumed the recommended amounts of both F&V according to their 24-h dietary recall. Additionally, our small sample of pre- and post-survey data showed the intervention group decreased their self-reported F&V consumption which may be more reflective of response bias than reality and corroborates the idea that children think they eat more than they actually do. This potential response bias to survey questions supports the use of 24-h dietary recalls and the use of mixed methods to determine dietary behavior [25].

Another explanation for the lack of F&V consumption in the US is that even though students and their parents report liking F&V, there could be barriers to accessing these foods (e.g., busy lifestyle, not living close to fresh produce) [34]. While this finding is consistent with prior research it further demonstrates the need for interventions that boost access to F&Vs for children.

### Limitations

Our study had several limitations. First, our small sample (*n* = 41), came from an independent school and existed of two naturally existing, not randomly assigned, groups. While the demographic survey completed by parents of students in each group indicate the intervention and control groups were of similar socioeconomic status and racial demographics, the intervention group was one grade older and consisted of more individuals (*n* = 25) than the control group (*n* = 16). It is also noteworthy that there was a low response rate (approximately 50%) on the parent demographic survey, which merits caution when comparing demographics between the intervention and control groups. While having an intervention and control group of different grade levels is a limitation, 10–12-year-olds (i.e., 5th and 6th graders) are sometimes grouped together developmentally, for example they are thought to have similar social interests [35], communication skills [35], and have the same recommendations for F&V consumption [33].

Second, the pre-curriculum survey found the intervention group had significantly more confidence cooking a meal. Theoretically, the control group and the intervention group should have had similar pre-survey results. No other statistically significant differences were seen between groups at baseline. While children aged 10–12 (i.e., 5th and 6th graders) are often grouped together developmentally [35], it is possible the intervention group (6th graders) have had more opportunities to cook than the 5th graders in the control group. Researchers recommend future studies modify this study designs to reduce potential bias stemming from differences in grade levels.

Third, the current study used the same pre/post curriculum survey questions that GHP has been using since the inception of the curriculum in 2017 which are proprietary and designed specifically to meet the evaluation aims of GHP (e.g., *Rate how you feel about eating vegetables from 1–5*). The survey questions used were not pulled directly from a validated survey tool.

Lastly, our findings for this study are limited to the specific curriculum model offered by GHP to this school, since that is what the students participated in. However, the GHP curriculum is robust, including several components like experiential learning, taste tests, and hands on work in school gardens, and it aligns with the Farm to School Network guidelines for F2S programming.

### Implications for research and practice

This study has implications for other F2S programs, the children and schools they serve, nutrition researchers, and policy makers. There is an alarming lack of F&V consumption in children in the US leading to a heavy burden of obesity and chronic disease. This study demonstrates promising impacts on children’s perceptions of F&V, which, according to prior research, may in turn influence consumption of F&V. Our findings corroborate the need for additional research to uncover clear connections between F2S programming and actual consumption of F&V.

F2S programs offer schools and the children they serve an engaging way to promote wellness. However, to have comprehensive and sustainable F2S programs policy makers like state departments of education and agriculture should continue providing funding for F2S, and school districts must invest in the infrastructure and staff needed to implement F2S programs.

## Data Availability

Not applicable.

## Footnotes

1 Two students arrived to class late and missed the rich picture activity.
